# Effect of video-assisted teaching on cyberbullying awareness among adolescents

**DOI:** 10.6026/973206300211380

**Published:** 2025-06-30

**Authors:** Rajendra D. Kharadi, Karthikadevi Mariappan, Jignasaben Patani, Mahalakshmi B., Gnanadesigan Ekambaram, Jothimani S., Jamunarani P., Nandha kumar S., Siva Subramanian N.

**Affiliations:** 1 Department of Forensic Medicine & Toxicology, Dr.N.D Desai Faculty of Medical Science and Research, Dharamsinh Desai University, Nadiad, Gujarat, India; 2 Department of Paediatric Nursing, Sri Ramachandra Faculty of Nursing, Sri Ramachandra Institute of Higher Education and Research, Porur, Chennai-600116, Tamil Nadu, India; 3 Department of Community Medicine, Nootan Medical College & Research Centre, Sankalchand Patel University, Visnagar, Gujarat, India; 4 Department of Paediatric Nursing, Nootan College of Nursing, Sankalchand Patel University, Visnagar, Gujarat, India; 5 Department of Physiology, Nootan Medical College and Research Centre, Sankalchand Patel University, Visnagar, Gujarat, India; 6 Department of Psychiatric Nursing, Hindustan college of Nursing, Chengalpattu, Chennai, Tamil Nadu, India; 7 Department of Psychiatric Nursing KMCH College of Nursing, Coimbatore-641048, Tamilnadu, India; 8 Department of Psychiatric Nursing, Nootan College of Nursing, Sankalchand Patel University, Visnagar, Gujarat - 384315, India

**Keywords:** Cyberbullying, adolescents, video-assisted teaching, knowledge enhancement, school-based intervention

## Abstract

Cyberbullying is a growing public health concern among adolescents, often leading to serious psychological consequences. Therefore, it is of interest to evaluate the effectiveness of video-assisted teaching (VAT) on improving cyberbullying awareness among adolescents in Mehsana district schools. A total of 60 students aged 15-18 years participated in a pre-experimental one-group pretest-posttest design. Pre-test results showed 48% of students had poor knowledge, which dropped to 10% post-intervention, with "good" knowledge rising to 60%. A significant increase in mean knowledge scores was observed (t = 9.58, p < 0.05). Among all demographic variables, only religion showed a significant association with pre-test knowledge. The study highlights VAT as a cost-effective and impactful tool for cyberbullying awareness in school settings.

## Background:

Cyberbullying has emerged as a significant public health issue, especially among adolescents who are frequent users of digital technology. It involves the use of electronic communication to bully, harass, or threaten individuals and can be more pervasive than traditional bullying due to its 24/7 nature and the anonymity it often provides. Adolescents, still developing emotionally and socially, are particularly vulnerable to the psychological harm cyberbullying can cause [1]. The alarming prevalence of cyberbullying among adolescents calls for urgent and effective educational interventions. In a study involving Indian adolescents, 90% reported either being victims or witnesses of cyberbullying, with a substantial number unaware of how to handle such situations. The study highlighted the lack of structured awareness programs and emphasized the importance of proactive teaching strategies in schools [2].

One effective strategy gaining traction is video-assisted teaching (VAT), which blends auditory and visual elements to facilitate deeper engagement and comprehension. VAT is particularly suitable for adolescents as it aligns with their multimedia-rich learning preferences and has shown promise in enhancing knowledge retention in health education settings [3]. Recent research on cyberbullying education demonstrates that video-based interventions can lead to significant improvements in awareness and behavior. In a pre-experimental study, students who participated in a video-assisted program showed a notable increase in knowledge, with mean scores rising dramatically post-intervention. This validates VAT as an impactful and scalable approach to adolescent education [4]. Beyond improving knowledge, VAT can influence social attitudes and behavioural intentions. In a video-based cyberbullying prevention program for college students, researchers found reductions in pro-bullying attitudes and actual bullying behavior up to one month after the intervention. Although changes in empathy were limited, the program still showed promise in curbing harmful behaviors over time [5]. Including parents in intervention strategies may further reinforce learning and behavioural changes. A recent web-based cyberbullying awareness program in Türkiye involved both adolescents and their parents and significantly increased their understanding of cyberbullying while reducing the incidence of such behaviors [6]. This indicates the value of family-centered education in amplifying the impact of school-based programs [7]. Therefore, it is of interest to evaluate the effectiveness of video-assisted teaching (VAT) on improving cyber-bullying awareness among adolescents in Mehsana district schools.

## Methodology:

## Research design and approach:

This study employed a quantitative pre-experimental one-group pretest-posttest design to assess the effectiveness of a video-assisted teaching program on cyberbullying knowledge among adolescents. This design allowed for measuring changes in knowledge levels before and after the intervention.

## Setting and Sample:

The study was conducted in three schools in Mehsana District, Gujarat, Utkarsh Vidyalaya, T.K. Vidyalaya and Urban Progressive Girls High School. A total of 100 adolescents aged 15-18 years were selected using a non-probability convenience sampling technique.

## Inclusion and exclusion criteria:

Participants were students from classes 11 and 12, able to read and write in Gujarati or English and willing to participate. Adolescents with previous experiences as victims of cyberbullying or unavailable during data collection were excluded.

## Tool and intervention:

A self-structured questionnaire consisting of 25 multiple-choice questions assessed knowledge across key areas such as definitions, forms, consequences and prevention of cyberbullying. Knowledge was categorized as poor (0-8), average (9-17), or good (18-25). The intervention was a short video in Gujarati, presented via projector, covering essential topics on cyberbullying awareness and prevention.

## Data collection and analysis:

The data collection occurred in three stages: pre-test, intervention (video-assisted teaching) and post-test after 7 days. Data were analyzed using descriptive statistics (mean, SD, frequency) and inferential statistics, including a paired t-test to assess intervention effectiveness and chi-square tests to explore associations with demographic variables.

## Ethical considerations:

Permission was obtained from school authorities and informed consent was taken from participants. Confidentiality and ethical standards were strictly maintained.

## Results:

Table 1 (see PDF) shows that most participants were aged 15-16 years (63.33%) equally split by gender and predominantly from the arts stream (66.66%). A majority came from joint families (50%) and middle to lower-income groups. While 58.33% had prior knowledge of cyberbullying, 76.66% had no direct experience. The most common source of information was friends (40%). Above graph shows a clear improvement in students' knowledge of cyberbullying after video-assisted teaching, with "good" knowledge levels rising from 6.66% to 60% and "poor" levels dropping from 48% to 10%. Table 2 (see PDF) shows that the mean knowledge score significantly increased after the intervention. The t-value of 9.58 with a p-value less than 0.05 confirms that the video-assisted teaching was statistically effective. Table 3 (see PDF) shows that among all the selected demographic variables, only religion had a statistically significant association with pre-test knowledge scores (p < 0.05). Other variables such as age, gender, standard and stream of study did not show any significant association, indicating that knowledge levels before the intervention were largely independent of these factors. Figure 1 (see PDF) shows a marked improvement in knowledge of post-test regarding cyberbullying as compared to pretest score and indicates video assisted teaching is effective in enhancing knowledge on cyberbullying among adolescents.

## Discussion:

The current study aimed to assess the effectiveness of video-assisted teaching on cyberbullying knowledge among adolescents in selected schools of Mehsana District. The findings clearly demonstrate the positive impact of video-based educational interventions in enhancing awareness and understanding of cyberbullying. Before the intervention, nearly half (48%) of the participants demonstrated poor knowledge regarding cyberbullying and only 6.66% showed good knowledge. This finding aligns with earlier studies indicating a widespread lack of cyberbullying awareness among school-aged adolescents. For instance, Singh *et al.* found that a video assisted knowledge regarding cyber-bullying among adolescents of selected higher secondary school [8]. The pre-test findings revealed that a large proportion (48%) of students had poor knowledge about cyberbullying. This result is in line with global studies highlighting that adolescents often lack sufficient awareness of digital risks. Gaffney *et al.* also reported that schools were underprepared to address cyberbullying due to limited teacher training and outdated disciplinary frameworks [9]. A more recent study by Kutok *et al.* emphasized that adolescents lack emotional and technical readiness to handle cyber-conflict on social media [10]. Post-intervention results demonstrated a dramatic rise in knowledge, with "good" scores increasing to 60%. The t-value of 9.58 indicated statistically significant improvement. These findings are consistent with studies showing that video-based teaching improves understanding and retention. Patidar and Asari showed a similar knowledge increase after video-assisted teaching in Mehsana district, with a mean post-test score of 16.02 compared to 9.5 in the pre-test [11], while Zod *et al.* confirmed educational value with a significant t-value of 19.98 in a pre-experimental study [12]. Doane *et al.* found measurable drops in bullying behavior and knowledge improvements after video intervention [13]. Sarasmita *et al.* further validated that digital media tools such as videos and serious games significantly enhance knowledge and behavior change outcomes in adolescents [14]. Vlaanderen *et al.* found that video interventions based on behavioral theories successfully reduced intentions to cyberbully and increased empathy [15]. Further evidence from Uludaşdemir and Küçük emphasized that web-based educational videos, when combined with parental involvement, can amplify learning outcomes and reduce cyberbullying behaviors [16]. Kutok *et al.* also observed reduced stress and increased bystander intervention after video intervention through Instagram [17]. This study found that only religion had a statistically significant association with pre-test knowledge, while variables like age, gender and standard were not significant. This is somewhat unique, as most literature reports mixed results on demographics. Wang and Zhou linked violent video game exposure to increased cyberbullying, suggesting content exposure might mediate demographic impacts [18]. Minullina emphasized that adolescents' coping strategies in cyberbullying situations were influenced by emotional maturity rather than age or gender alone [19], while Kee *et al.* found peer social interaction more predictive of cyberbullying behavior than demographic characteristics (reference not listed - needs correction). Reason *et al.* emphasized the unique context of rural schools, where awareness gaps are larger due to fewer digital literacy programs [20], while Carter and Wilson also found no significant demographic link with cyberbullying, affirming current study findings [21]. The effectiveness of video-assisted learning across multiple international contexts confirms its scalability. Studies by Camelford and Ebrahim in the U.S. [22] and Cao in China [23], demonstrate its effectiveness in diverse socio-cultural settings. Dinakar *et al.* introduced a common-sense-based video interface to reflect on cyberbullying, noting its potential in empathy-building [24]. Hou reinforced the value of school-based multimedia interventions to reduce harm and increase awareness [25].

## Conclusion:

Video-assisted teaching significantly enhances adolescents' knowledge of cyberbullying. It serves as an effective, engaging and scalable educational method for school-based interventions. Integrating such approaches into curricula can empower youth and foster safer digital environments.
